# Randomized Trial of Postoperative Radiation Therapy After Wide Excision of Neurotropic Melanoma of the Head and Neck (RTN2 Trial 01.09)

**DOI:** 10.1245/s10434-024-15569-2

**Published:** 2024-06-08

**Authors:** Mark B. Pinkham, A. Herschtal, A. M. Hong, M. S. -T. Chua, R. A. Scolyer, S. Cumming, A. Pullar, J. Nobes, C. A. Barker, B. A. Guadagnolo, G. B. Fogarty, B. H. Burmeister, M. C. Foote

**Affiliations:** 1 Department of Radiation Oncology, Princess Alexandra Hospital, Brisbane, Australia; 2 University of Queensland, Brisbane, Australia; 3 School of Public Health and Preventive Medicine, Monash University, Melbourne, Australia; 4 Department of Radiation Oncology, Chris O’Brien Lifehouse, Sydney, Australia; 5 Melanoma Institute Australia, The University of Sydney, Sydney, Australia; 6 Genesiscare, Mater Hospital, North Sydney, Australia; 7 Department of Radiation Oncology, Peter MacCallum Cancer Centre, Melbourne, Australia; 8 The Sir Peter MacCallum Department of Oncology, The University of Melbourne, Melbourne, VIC Australia; 9 Department of Tissue Pathology and Diagnostic Oncology, Royal Prince Alfred Hospital and NSW Health Pathology, Sydney, Australia; 10 Faculty of Medicine and Health, The University of Sydney, Sydney, Australia; 11 Charles Perkins Centre, The University of Sydney, Sydney, Australia; 12 Melanoma and Skin Cancer Research Centre, Monash University, Melbourne, Australia; 13 Queensland University of Technology, Brisbane, Australia; 14 Norfolk and Norwich University Hospital NHS Foundation Trust, Norwich, UK; 15 Department of Radiation Oncology, Memorial Sloan Kettering Cancer Center, New York, NY USA; 16 Department of Radiation Oncology, MD Anderson Cancer Centre, Houston, TX USA; 17 ICON Cancer Centre, Revesby, Australia; 18 GenesisCare Fraser Coast, Hervey Bay, Australia

**Keywords:** Neurotropic, Desmoplastic, Cutaneous melanoma, Head & neck, Radiation therapy, Randomised trial

## Abstract

**Background:**

Cutaneous neurotropic melanoma (NM) of the head and neck (H&N) is prone to local relapse, possibly due to difficulties widely excising the tumor. This trial assessed radiation therapy (RT) to the primary site after local excision.

**Methods:**

Participants from 15 international centers were randomized to observation or RT. The participants were required to have microscopically negative excision margins 5 mm wide or wider and no evidence of disease elsewhere. The primary outcome was time to local relapse. The secondary outcomes included time to any recurrence, overall survival (OS), and toxicity.

**Results:**

The trial ceased prematurely due to slow recruitment and the COVID-19 pandemic. During 2009–2020, 50 participants were randomized: 23 to observation and 27 to RT. The most common NM subsites were scalp (32%), midface (22%), and lip (20%). The median depth of invasion was 5 mm, and desmoplasia observed in 69%. The median duration from randomization to last contact was 4.8 years. Four participants (8%) experienced local relapse as a first recurrence during the study period: 3 in the observation arm and 1 in the RT arm (hazard ratio [HR] 0.29; 95% confidence interval [CI] 0.03–2.76; *p* = 0.279). No statistically significant difference in time to any relapse or OS was observed. More than 6 months after randomization, grade 3 or greater toxicity was experienced by 10% of the participants in the observation arm and 12.5% of the participants in the RT arm of the study.

**Conclusion:**

Due to low accrual, the role of adjuvant RT for cutaneous NM of the H&N excised with microscopically negative margins 5 mm wide or wider remains undefined. Its routine use cannot be recommended. Local relapse might be less common than previously anticipated based on retrospective reports.

**Supplementary Information:**

The online version contains supplementary material available at 10.1245/s10434-024-15569-2.

Neurotropic melanoma (NM) is a rare subtype of cutaneous melanoma characterized by melanoma cells surrounding neural structures (perineural invasion) within nerve sheaths (intraneural invasion) or exhibiting neural differentiation. Desmoplasia is typically seen in association with NM, but non-desmoplastic tumors with neurotropism also occur.^1,2^

Compared with other melanoma subtypes, desmoplastic melanoma and NM are more commonly amelanotic and located on the head and neck (H&N) region of older men.^1–3^ These lesions have a propensity to be thicker at diagnosis, and have been associated with an elevated risk of local recurrence after surgery.^1–14^ Recurrence involving large and/or cranial nerves can be associated with significant neurologic morbidity.^15^ The incidence of nodal involvement may be lower for NM than for non-NM, but conventional prognostic factors including primary thickness, mitotic rate, ulceration, male sex, older age, and stage of disease still apply.^2,10,15–17^

The preferred management of localized NM involves excision with wide margins.^4,10,18^ In the H&N, this may not always be achievable with preservation of function and cosmesis given their proximity to sensitive tissues and important anatomic structures. Moreover, the amelanotic and infiltrative tumor morphology may make clinical or pathologic assessment of extent challenging. Margins smaller than 1 cm or unknown size have been correlated with an increased risk for local recurrence of desmoplastic melanoma and NM after surgery.^2,3,6,7,10–12^ Many retrospective studies have suggested that adjuvant radiation therapy (RT) may reduce this risk^2,3,7–9,11,19^ although randomized studies are lacking. The NCCTG N0275 (Alliance) prospective single-arm study recruited 20 patients with desmoplastic melanoma to receive adjuvant RT to 30 Gy in five fractions after wide local excision. The authors concluded that the treatment was efficacious and well-tolerated.^20^

We performed a randomized controlled trial to assess the role of RT administered to the primary tumor site after excision for localized cutaneous NM of the H&N region.

## Methods

This research was approved by the Metro South Health Human Research Ethics Committee (HREC/11/QPAH/272) and prospectively registered with ClinicalTrials.gov (NCT00975520) and the Australian and New Zealand Clinical Trials Registry (ACTRN12611000212954).

For inclusion in the study, a NM located above the jaw/occiput (head primary) or between the jaw/occiput and clavicles (neck primary) was necessary. The extent or degree of neurotropism required for eligibility was not specified, but the study excluded those with named cranial or cervical nerve involvement clinically or on magnetic resonance imaging (MRI).

Initial surgery required complete macroscopic removal of all visible disease with a clinical margin 1 cm wide or wider (when practical) and at least a 5-mm microscopically negative margin unless constrained by an anatomic boundary. No evidence of residual perineural, in-transit, nodal, or metastatic spread was permitted, as determined by clinical examination, sentinel lymph node biopsy, elective nodal dissection, or any form of imaging.

The participants were required to be at least 18 years old and have an ECOG performance status of 0 to 2. The study excluded those with a history of cancer (unless treated ≥5 years earlier or treated for cutaneous melanomas in situ ≥2 years earlier, with no evidence of recurrence) or prior RT to the H&N region.

Eligible participants were randomized 1:1 using an online system with stratification for institution and anatomic location (head vs neck primary). All the participants underwent central pathology review to confirm eligibility, but not all were reviewed before randomization.

The primary outcome was time from randomization to local relapse. Local relapse was defined as recurrent disease within the tumor bed, defined as a 2-cm perimeter surrounding the excision scar (excluding any surgery for local flap). The limits of the tumor bed were demarcated on the skin of all the participants before randomization, and a clinical photograph was taken. Death due to any cause, regional relapse, and distant relapse were considered censoring events.

The secondary outcomes were time from randomization to any relapse, overall survival (OS), cancer-specific survival (CSS), late toxicity, and quality of life (QoL). Follow-up evaluation comprised clinical examination and QoL assessment at baseline after surgery, then every 3 months for the first 2 years, and finally every 6 months until 5 years. Surveillance computed tomography (CT) imaging of the head, neck, and chest was performed annually for 5 years, and MRI of the region of interest also was mandated when the tumor bed lay directly above a named nerve.

Clinical management of recurrent disease at any stage was according to the standard of care per local treating physician discretion. Toxicity was recorded according to the Common Terminology Criteria for Adverse Events (CTCAE version 4.0). Acute toxicity was defined as toxicity 0 to 3 months after randomization and also assessed 2 and 6 weeks after RT. Late toxicity was defined as anything 6 months or longer after randomization. Quality of life was assessed using the EORTC QLQ-C30 (version 3.0) and QLQ-H&N35 (version 1) modules.^21,22^

Radiation therapy was to begin within 12 weeks (and not more than 14 weeks) after surgery. A planning CT was mandated to determine target volumes and for dose calculation purposes. The surgical bed was defined on the CT as the region incorporating excision scar, cavity, and deep tissues (excluding any local flap) down to the next uninvolved tissue layer. The RT treatment volume was defined as a 1.5-cm expansion of the surgical bed in all directions, adjusted to anatomic boundaries such as the skin surface and bone. The prescribed dose was 48 Gy in 20 fractions at five per week.^23^ All the participants receiving RT underwent central RT quality assurance review after treatment. Additional technical information regarding RT technique and dose reporting is provided in the Supplementary Material.

A pragmatic accrual goal of 100 participants was determined, with follow-up evaluation for 5 years after randomization. This was intended to yield 80% power to detect an 18% difference in recurrence rates between the study arms based on historical cohorts indicating an anticipated local relapse rate of 35% at 3 years in the control arm.^9,10,14^

For time-to-event analyses, survival was characterized per arm using the Kaplan-Meier method, and Cox proportional hazards regression modeling was used to determine hazard ratios (HRs) and 95% confidence intervals (CIs). For QoL, each module was analyzed separately, with mean scores taken for each participant at every assessment time point. The scale direction was reversed when necessary to ensure that a higher score reflected better health-related QoL. For QLQ-C30, the five functional subscales, eight symptom subscales, and two global health questions were considered separately. For QLQ-H&N35, the pain subscale was used.

To compare changes in QoL from baseline between arms, a linear mixed-effects model was constructed. Differences between the arms were assessed statistically using the area under the QoL-time curve. A *p* value lower than 0.05 was considered statistically significant.

## Results

**Fig. 1:**
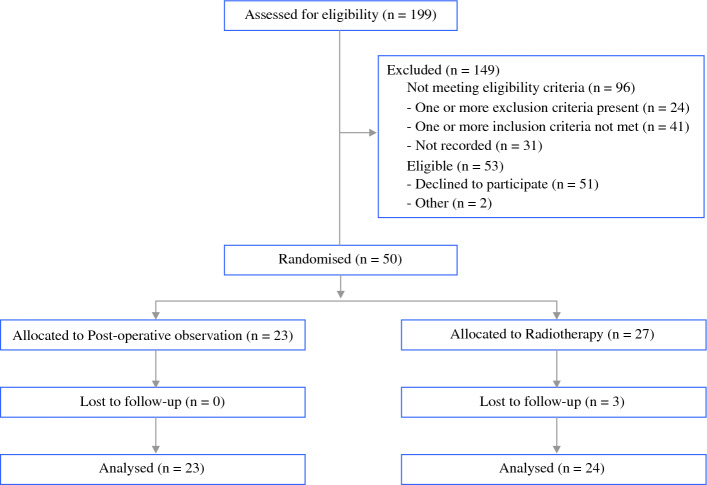
CONSORT diagram of participant flow

Recruitment began in 2009. Due to slow accrual and the COVID-19 pandemic, the Data Safety Management Committee recommended that the trial close prematurely in December 2020. At this time, the trial had 50 participants: 23 allocated to the observation arm and 27 to the RT arm (Fig. 1).

At registration, the median age of the participants was 68 years (range 29–90 years), 84% were male, and 96% had an ECOG performance status of 0 or 1. The most common primary sites were scalp (32%), midface (22%), and lip or chin (20%). The median tumor thickness was 5 mm (range 1.1–15.0 mm), and 68% had Clark level V disease. In 69% of the participants, the NM had an associated desmoplastic melanoma component. The median pathologic excision margin clearance was 9.5 mm (range 0.1–45.7 mm) laterally and 2.5 mm (range 0.1-13.0) deep. Additional baseline characteristics are summarized in Table 1. The median duration from randomization to the last contact was 4.8 years in both arms. Central pathology review confirmed that all participants were eligible. No participants received systemic therapy of any type for melanoma before the first recurrence.

**Table 1:** Baseline characteristics^a^

	Observation	Radiotherapy	Overall
	(*n* = 23) *n* (%)	(*n* = 27) *n* (%)	(*n* = 50) *n* (%)
*Primary site*			
Forehead or temple	2 (8.7)	4 (14.8)	6 (12.0)
Midface	4 (17.4)	7 (25.9)	11 (22.0)
Postauricular	0	1 (3.7)	1 (2.0)
Lip or chin	4 (17.4)	6 (22.2)	10 (20.0)
Scalp	9 (39.1)	7 (25.9)	16 (32)
Neck	4 (17.4)	2 (7.4)	6 (12)
*Primary thickness* (mm)			
Median (range)	5.0 (1.1–15.0)	5.2 (2.0–14.0)	5.0 (1.1–15.0)
*Clark level*			
III	1 (4.5)	0	1 (2.1)
IV	7 (31.8)	5 (20)	12 (25.5)
V	12 (54.5)	20 (80)	32 (68.1)
Unknown	3 (13.0)	2 (7.4)	5 (10)
*Perineural invasion*			
Unifocal	5 (22)	8 (30)	13 (26)
Multifocal	9 (39)	10 (37)	19 (38)
Unknown	9 (39)	9 (33)	18 (36)
*Largest involved nerve diameter* (mm)			
Median (range)	0.1 (0.05–0.2)	0.07 (0.04–4)	0.1 (0.04–4.0)
Unknown	17 (74)	14 (52)	31 (62)
*Ulceration*			
Present	8 (34.8)	5 (18.5)	13 (26.0)
*Microsatellites*			
Present	1 (4.3)	1 (3.7)	2 (4.0)
*Desmoplasia*			
Present	13 (56.5)	21 (80.8)	34 (69.4)
*Mitotic number* (per mm^2^)			
Median (range)	3.5 (0.0–18.0)	3.0 (1.0–26.0)	3.0 (0.0–26.0)
*Excision margin clearance* (mm)			
Lateral: median (range)	7.0 (0.1–45.0)	10.0 (0.3–45.7)	9.5 (0.1–45.7)
Deep: median (range)	3.0 (0.1–13.0)	1.2 (0.1–11.0)	2.0 (0.1–13.0)
Missing	5 (23)	3 (11)	8 (16)

^a^Midface includes cheek, nose, pinna, preauricular

**Fig. 2:**
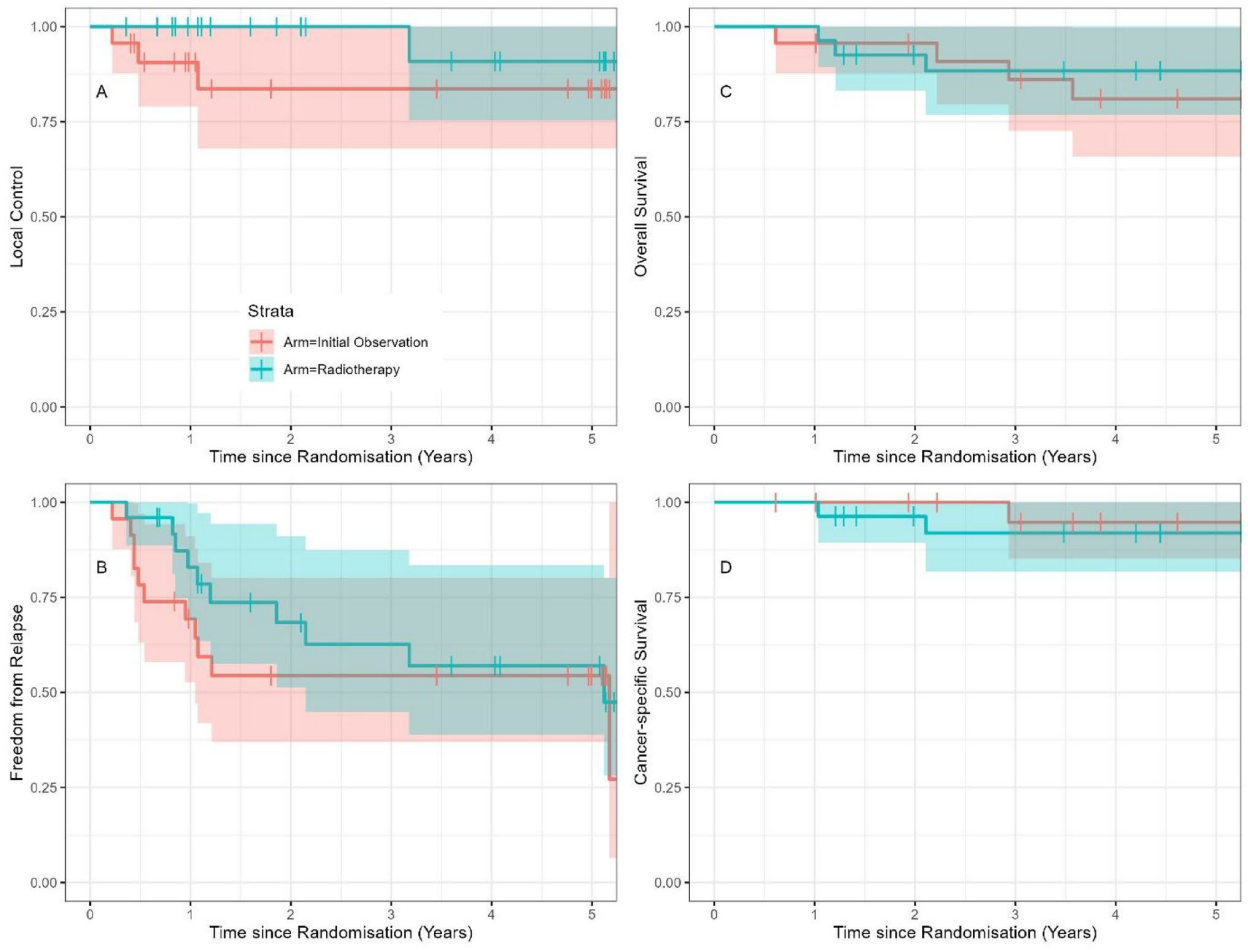
Kaplan-Meier product limit curves for **A** time to local relapse, **B** time to any relapse, **C** overall survival, and **D** cancer-specific survival according to treatment arm

Four participants (8%) experienced local relapse as their first recurrence during the study period: three (13%) in the observation arm and one (4%) in the RT arm (HR 0.29; 95% CI 0.03–2.76; *p* = 0.279; Fig. 2A). In the observation arm, these episodes occurred at 0.2, 0.5, and 1.1 years, and the only one in the RT arm occurred at 3.2 years. Four participants had multiple recurrences during the study period, including two additional local relapses as later events (Table 2). Thus, six episodes of local relapse occurred during the study period: four in the observation arm and two in the RT arm.

**Table 2:** Patterns of recurrence in participants who experienced recurrence during the study period

Recurrence pattern	Observation	Radiotherapy	Overall
Single: local	2	1	3
Single: regional	3	2	5
Single: distant	4	5	9
Multiple: L-L	1	0	1
Multiple: L-D	1	0	1
Multiple: R-R-D-L	0	1	1
Multiple: D-D	0	1	1

L, local; D, distant; R, regional

The most common treatment approach for the patients with the first isolated local relapse was re-excision followed by adjuvant RT. In these cases, the ultimate rate of local control after salvage therapy remained high. Further details are summarized in Table S1. Key histopathologic features of the primary NM in these participants are summarized in Table 3.

**Table 3:** Select histopathologic features of all neurotropic melanomas (NMs) with local recurrence during the study period

ID	Arm	Site	Thickness (mm)	Desmoplasia	Closest margin (mm)	PNI (largest diameter)
RTN-031	Obs	Lip	8	Present	10	Multifocal (0.2 mm)
RTN-040	Obs	Scalp	3	None	3.4	Unknown
RTN-046	Obs	Neck	15	Present	2.8	Unknown
RTN-047	Obs	Neck	4.6	None	1.1	Multifocal (0.1 mm)
RTN-006	RT	Lip	7	Present	5	Unknown
RTN-009	RT	Lip	3.8	Present	5	Multifocal (unknown)

*PNI* perineural invasion; *Obs* observation; *RT* radiation therapy

The most common recurrence pattern overall was distant, with four in the observation arm and five in the RT arm. No local relapses occurred within 30 days after a first relapse of another type.

Kaplan-Meier product limit curves were generated for time to any relapse, OS and CSS according to treatment arm (Fig. 2B–D). Comparing observation with RT, the HR for time to any recurrence, OS and CSS were 0.71 (95% CI 0.30–1.68; *p* = 0.441), 0.65 (95% CI 0.14–2.89; *p* = 0.569), and 1.72 (95% CI 0.16–19.05; *p* = 0.656), respectively. Three participants with no follow-up assessments were omitted from all recurrence-based time-to-event analyses, but still were included in the analyses for OS and CSS.

Toxicity was assessed in both arms according to type at three time points: after surgery (*n* = 50), 0 to 3 months later (acute toxicity) (*n* = 26), and after 6 months (late toxicity) (*n* = 37). Missing or incomplete data were noted disproportionately in the observation arm. At baseline after surgery, the highest toxicity was grade 0 or 1 in 88%, grade 2 in 8.2%, and grade 3 or 4 in 4.1% of the participants. No participants experienced acute toxicity (grade ≥3) in either arm. Grade 3 late toxicity was experienced by 10% of the participants in the observation arm and 12.5% of the participants in the RT arm. Table 4 demonstrates the late toxicity types observed per participant for each arm. Given the overall low incidence of grade ≥3 late toxicity observed, planned statistical comparisons between the arms were not performed.

**Table 4:** Late (≥6 months from randomization) toxicity grades per toxicity type and highest grade per individual

	Observation arm	Radiotherapy arm	Overall
	(*n* = 13) *n* (%)	(*n* = 24) *n* (%)	(*n* = 37) *n* (%)
*Skin*			
0–2	10 (100)	24 (100)	34 (100)
≥3	0	0	0
Missing	3 (23.1)	0	3 (8.1)
*Subcutaneous tissue*			
0–2	9 (100)	23 (95.8)	32 (97)
≥3	0	1 (4.2)	1 (3)
Missing	4 (30.8)	0	4 (10.8)
*Mucous membrane*			
0–2	7 (100)	24 (100)	31 (100)
≥3	0	0	0
Missing	6 (46.2)	0	6 (16.2)
*Salivary gland*			
0–2	7 (100)	24 (100)	31 (100)
≥3	0	0	0
Missing	6 (46.2)	0	6 (16.2)
*Nerve damage*			
0–2	8 (88.9)	23 (95.8)	31 (93.9)
≥3	1 (11.1)	1 (4.2)	2 (6.1)
Missing	4 (30.8)	0	4 (10.8)
*Inner ear hearing*			
0–2	9 (100)	23 (95.8)	32 (97)
≥3	0	1 (4.2)	1 (3)
Missing	4 (30.8)	0	4 (10.8)
*Highest grade per participant*			
0–2	9 (90)	21 (87.5)	30 (88.2)
≥3	1 (10)	3 (12.5)	4 (11.8)
Missing	3 (23.1)	0	3 (8.1)

**Fig. 3:**
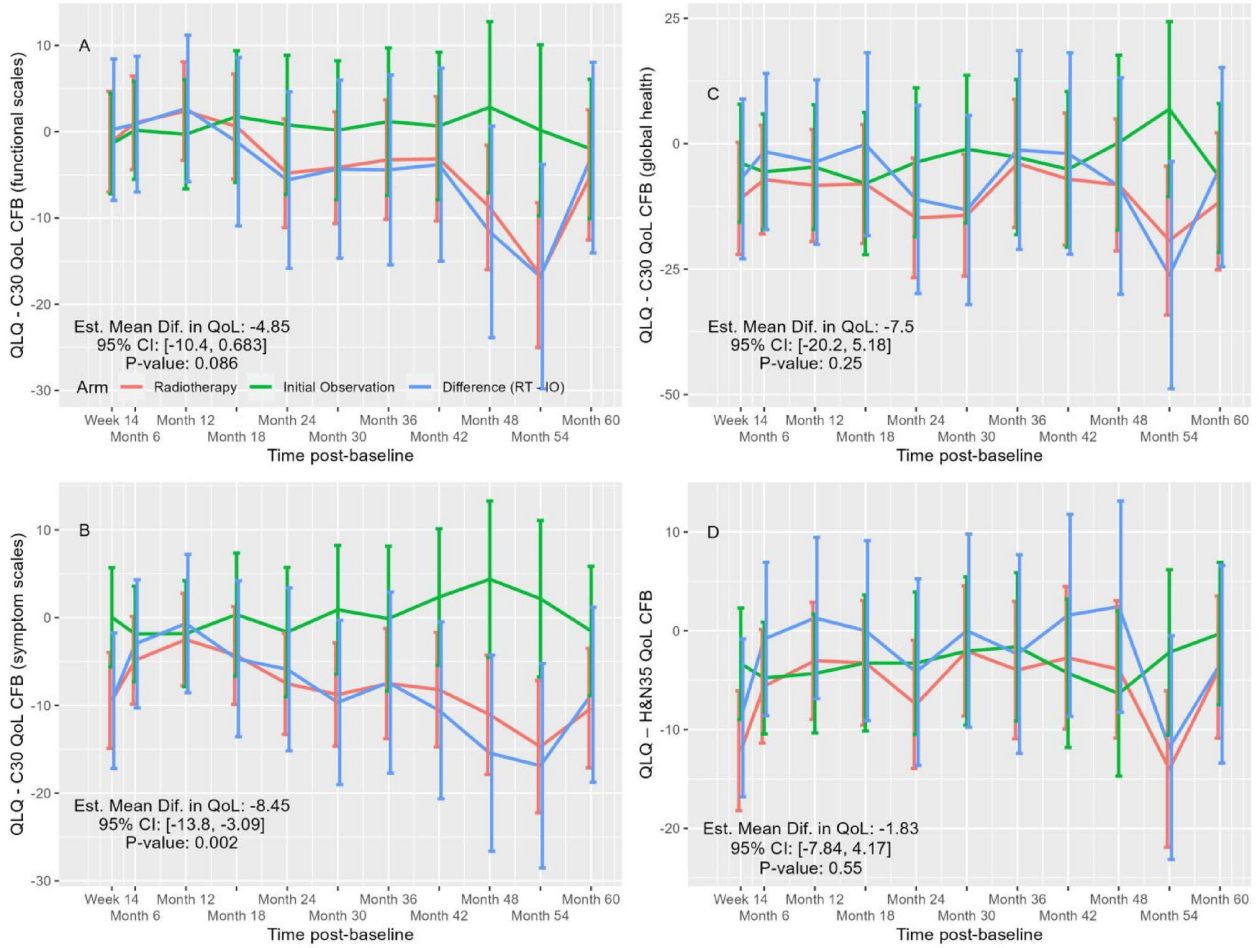
Changes in quality of life (QoL) over time for participants allocated to adjuvant radiotherapy and observation arms according to **A** EORTC QLQ-C30 functional subscales, **B** symptom subscales, **C** global health subscales, and **D** EORTC QLQ-H&N35 pain subscale. Higher scores indicate better QoL

Changes in QoL from baseline favored observation and persisted until 5 years after randomization, with a statistically significant difference observed only in the symptom subscales (Fig. 3A–D). By month 60 at the end of the study, the proportion of participants with complete QoL data was 48% in the observation arm and 37% in the RT arm. Low QoL assessment completion rates were mostly attributable to disease recurrence. Comparing RT with observation, the mean difference in QoL score change from baseline between the arms using the EORTC QLQ-C30 module was − 4.85 (95% CI − 10.40 to 0.68; *p* = 0.086) for functional subscales, − 8.45 (95% CI − 13.8 to 3.09; *p* = 0.002) for symptoms subscales, and − 7.45 (95% CI –20.17 to 5.18; *p* = 0.25) for global health. For the EORTC QLQ-H&N35 module pain scale, it was − 1.84 (95% CI − 7.84 to 4.17; *p* = 0.55).

## Discussion

This trial is the only randomized study of adjuvant RT for cutaneous NM of the H&N. Consistent with prior retrospective studies, the participants randomized to adjuvant RT after excision experienced local relapse less frequently than those randomized to initial observation, but the difference was not statistically significant. The rate of local relapse observed in the control arm was much lower than expected. Combined with incomplete accrual, the trial was therefore underpowered for the primary end point.

Several factors may have accounted for the observed lower than anticipated risk of local relapse after excision alone. First, the participants may have undergone more extensive surgery than historic cohorts. Most excisions or subsequent re-excisions were performed in large academic centers in which trial recruitment occurred. As illustrated in Table 1, some of the trial participants entered the trial with very wide peripheral excision margins. Similar findings were reported by Chen et al.^3^ in a large contemporary retrospective study of 128 patients with NMs, including 51% H&N primaries. Among those undergoing surgery alone, the rate of local relapse was only 6% and associated with positive margins as well as an H&N primary location.

Second, a microscopically negative excision pathologic margin of at least 5 mm was required for study inclusion unless constrained by an anatomic boundary. Prior clinical trials of a wide excision for cutaneous melanoma have typically required a clinical margin of at least 1 to 2 cm, but not a microscopic pathologic margin. This requirement may have biased the study cohort to participants with wider excision margins than usual.

Third, a centralized histopathology review was mandated in the trial to confirm negative margin status. Desmoplastic melanoma and NM are challenging to diagnose,^24^ and the incidence of microscopically positive margins may have been underestimated in historic cohorts.

Fourth, neurotropism may not be as compelling a risk factor for local recurrence as previously described. Most older studies failed to distinguish between pure NM and NM with a desmoplastic component because these features commonly coexist. Varey et al.^2^ reported on a cohort of 1389 patients with cutaneous melanoma, including 671 patients with NM (72% mixed with desmoplasia and 28% without desmoplasia). When control was used for other factors, the presence of neurotropism was not associated with risk of local recurrence.

Finally, clinician and/or participant equipoise may have changed during the trial period, as demonstrated by the significant proportion of eligible patients in Fig. 1 who elected not to participate. The impact of the role emerging for immunotherapy in this population during the study period is uncertain.^25^

Qualitative assessment of histopathologic risk factors for local relapse is limited by the small numbers involved. Lip primaries may be over-represented in Table 3, but no association with NM tumor thickness, co-existing desmoplasia, or closest excision margin is apparent. All local relapses with data available exhibited multifocal perineural invasion (PNI). Insufficient information precludes comment on the influence of the diameter of the largest nerve involved. In the multivariate analysis of 671 NMs by Varey et al.,^2^ excision margins of 8 mm or wider were associated with a fourfold lower risk of local relapse than those of 2 mm or smaller (HR 0.24; *p* < 0.001). Moreover, when excision margins were smaller than 8 mm in their cohort, adjuvant RT halved the risk of local relapse (HR 0.48; *p* = 0.02). Based on these data, the current Australian Cancer Council guidelines recommend consideration of RT for all patients with desmoplastic melanoma or NM for whom excision margins of 8 mm or wider cannot be achieved,^18^ and the United States National Comprehensive Cancer Network guidelines recommend considering RT at the site of resected primary tumor for selected patients at high risk based on desmoplastic histology and/or neurotropism.^26^

Toxicity did not differ significantly between the arms of the study. After adjuvant RT, acute toxicity did not exceed grade 2 in any patients, and the rate of grade ≥3 late toxicity was comparable with that for patients receiving surgery alone. The mean changes in QoL from baseline favored the observation arm and ranged from − 8.5 to − 1.8 points across a variety of subscales. However, these are expected to be of little clinical significance because minimally important differences (MIDs) are generally in the range 5 to 10 points, with deteriorations in the range 10 to 15 points or more considered medium and large.^27–29^ Disease-specific MIDs are estimated to be − 4 to − 12 for people with H&N cancer and − 5 to − 15 for patients with melanoma.^30^

Caution is required when the QoL data from the current study are interpreted due to the small numbers involved and the attrition of available data observed over time, as reflected in the wide confidence intervals presented. The proportion of patients with missing QoL data is similar to that reported in other randomized studies of comparable populations.^31,32^

In conclusion, the role of adjuvant RT for cutaneous NM of the H&N excised with widely negative pathologic margins (defined as ≥5 mm microscopically negative unless constrained by an anatomic boundary) remains undefined. Based on this study, the routine use of adjuvant RT to the primary site cannot be recommended. The rate of local relapse might be less common than previously anticipated. For patients experiencing isolated local relapse after initial wide excision and observation, the ultimate rate of local control after repeat surgery and adjuvant RT remains high. If pathologic margins are smaller than 5 mm and further excision is not feasible, adjuvant RT remains justifiable based on multiple retrospective series suggesting improved local control in this setting.^2,3,7,9^

### Supplementary Information

Below is the link to the electronic supplementary material.

Supplementary file1 (DOCX 19 KB)

Supplementary file2 (DOCX 13 KB)
