# Supplementary material for: Randomized Trial of Postoperative Radiation Therapy After Wide Excision of Neurotropic Melanoma of the Head and Neck (RTN2 Trial 01.09)
Source: Ann Surg Oncol. 2024 Jun 8;31(9):6088–96. doi: 10.1245/s10434-024-15569-2 (PMC11300506; doi:10.1245/s10434-024-15569-2)
Supplement: Supplementary file 1 — Supplementary file1 (DOCX 19 KB) [file 10434_2024_15569_MOESM1_ESM.docx]

**Supplementary Material**

Radiation therapy technique

1. Planning and simulation

Pre-treatment dental assessment was recommended in all dentate patients where the radiation therapy (RT) field is close to upper and lower teeth.

All patients were positioned supine and immobilised using either a thermoplastic mask or a vacuum formed mask. In cases where the posterior scalp vertex required RT, prone positioning was permitted if required.

All patients underwent planning computed tomography (CT) scan to aide target volume delineation and dose calculation. Surgical scars were marked (e.g. with radio-opaque wire). The entire volume of interest was scanned, preferably with 2.5 mm slice separation but no more than 5 mm.

1. Target volume definition

The following target volumes were contoured on the planning CT for all patients:

- Surgical bed volume (SBV); the volume of tissue incorporating the excisional scar (excluding scar from any local flap), visible surgical cavity and any deep tissues down to the next uninvolved tissue layer (e.g. muscle, bone, fat etc).
- Clinical target volume (CTV); SBV plus 1.5 cm expansion in all directions, cropped at anatomical boundaries such as skin surface and bone.
- Planning target volume (PTV); CTV plus 5 mm expansion in all directions.
- Planning target volume for evaluation (PTV_EVAL); PTV excluding any extension beyond the external skin contour into air and/or bolus. PTV_EVAL was only used for dosimetric assessment and not beam aperture design.

1. Dose prescription

The prescribed dose was 48 Gy in 20 fractions, daily fractionation over four weeks. In the event of missed treatment(s), the additional fractions were added to the end of the schedule.

1. Treatment technique

Bolus was applied to achieve the prescribed dose on the skin, as required.

Treatment with 3D-conformal RT, intensity-modulated RT (IMRT) or electrons was permitted. This determination was made by local investigators, applying principles set out in the protocol including requirements for PTV coverage.

1. Treatment planning objectives

For photon-based radiation therapy, the ‘near-minimum dose’ to PTV or PTV_EVAL was expected to be D98% ≥45.6 Gy (95% of the prescribed dose) and the ‘near-maximum dose’ to PTV or PTV_EVAL was expected to be D2% ≤51.36 Gy (107% of the prescribed dose).

For electron-based radiation therapy, the dose variation across PTV or PTV_EVAL was required to not exceed ±10% and the minimum dose to PTV or PTV_EVAL was expected to be 43.2Gy (encompassing the 90% isodose).

1. Maximum doses to normal tissues

Maximum doses to normal tissues were specified in the protocol for the following organs at risk. Additional constraints could be applied at the discretion of the local treating physician.

|  | Constraint |
| --- | --- |
| Spinal cord | Maximum ≤ 40 Gy |
| Eye lens | Maximum ≤8 Gy |
| Lacrimal gland | V40 Gy <50% |
| Parotid glands | At least one parotid must be spared to mean < 20Gy |

1. Radiation therapy quality assurance

Sites wishing to employ IMRT were required to undergo additional credentialing activities prior to study commencement.

All participants receiving RT underwent central RT quality assurance review after treatment.
