# Supplementary material for: Randomized Trial of Postoperative Radiation Therapy After Wide Excision of Neurotropic Melanoma of the Head and Neck (RTN2 Trial 01.09)
Source: Ann Surg Oncol. 2024 Jun 8;31(9):6088–96. doi: 10.1245/s10434-024-15569-2 (PMC11300506; doi:10.1245/s10434-024-15569-2)
Supplement: Supplementary file 2 — Supplementary file2 (DOCX 13 KB) [file 10434_2024_15569_MOESM2_ESM.docx]

**Supplementary Table 1. Treatment of individual local relapses during the study period.**

| **ID** | **Arm** | **Site** | **Relapse** | **Clinical management of local relapse** |
| --- | --- | --- | --- | --- |
| RTN-031 | Obs | Lip | L-L | 1^st^; surgery then adjuvant RT. 2^nd^; systemic therapy |
| RTN-040 | Obs | Scalp | L-D | Surgery |
| RTN-046 | Obs | Neck | L | Surgery then adjuvant RT |
| RTN-047 | Obs | Neck | L | Surgery then adjuvant RT |
| RTN-006 | RT | Lip | R-R-D-L | Systemic therapy |
| RTN-009 | RT | Lip | L | Surgery then adjuvant RT |

Obs= observation; RT= radiation therapy; D= distant; R= regional; L= local
